# Ballistic Behavior of Oblique Ceramic Composite Structure against Long-Rod Tungsten Projectiles

**DOI:** 10.3390/ma12182946

**Published:** 2019-09-11

**Authors:** Dujun Luo, Yangwei Wang, Fuchi Wang, Huanwu Cheng, Yu Zhu

**Affiliations:** 1 School of Materials Science and Engineering, Beijing Institute of Technology, Beijing 100081, China; luodujun@bit.edu.cn (D.L.); wangfuchi@bit.edu.cn (F.W.); chenghuanwu@bit.edu.cn (H.C.); 1055480577@163.com (Y.Z.); 2 National Key Laboratory of Science and Technology on Material under Shock and Impact, Beijing 100081, China

**Keywords:** ceramic armor, silicon carbide, penetration, oblique impact

## Abstract

Oblique ceramic armor structure composed of an oblique part and a backing part was designed to resist the ballistic impact of long rod penetrators. The front part consisted of an oblique silicon carbide ceramic and a triangular titanium alloy prism. The backing part contained layered silicon carbide and armor steel designed to absorb the residual energy of penetrators. The structure’s response to penetration was examined experimentally by considering different impact locations on oblique targets. Numerical simulations of the experiments were performed to reproduce the penetration and failure processes that occurred in the armor modules. In addition, a simple layer structure with the identical line-of-sight thickness of each material used in the oblique impact was simulated under a normal impact. The rod and target performances with the oblique impact and normal impact were compared and analyzed in detail. The results showed that the oblique structure had a better ballistic performance as a result of an extra short dwell period before penetrating the ceramic in comparison with the normal layer case. The ability of the oblique targets to defeat long rod projectiles differed with the impact location on the ceramic. The present study paves the way for ceramic armor obliquity applications.

## 1. Introduction

The interaction of oblique metallic materials subjected to long rod impact has been extensively studied for many years with the goal of dispersing the pressure derived from the impact or even deflecting or fracturing the projectile [1,2,3,4,5]. However, most of the studies on ceramic armor structures against long rod impact have focused on the normal impact case [6,7,8,9]. The influence of a ceramic target’s obliquity on its ballistic resistance has rarely been investigated because the inner mechanism is complex. Early work to assess the effect of ceramic target obliquity was mainly based on mass efficiency factors (MEFs) [10,11] or theoretical analyses [12,13,14], until the dwell mechanism for ceramic materials was proposed by Hauver and his colleagues [15]. This phenomenon occurs when a high-velocity projectile impacts a ceramic target and flows out radially along the surface of the ceramic [16]. No significant penetration in the ceramic after impact was termed as completely dwell or interface defeat.

Over time, new techniques have been developed such as rigid lateral confinement [17,18,19,20,21], pre-stress constraint [22,23,24], and the addition of cover materials [25,26,27,28,29] to ceramics, which have been proven to be reasonable ways to improve the dwell performance of ceramics under the normal impact condition. However, it was found that a copper-covered ceramic did not achieve a good ballistic resistance to an oblique impact [30], with better results obtained for a bare oblique target (no cover used) [31]. Compared with a normal impact, a radial asymmetric crack pattern linked with the buffer layers was considered to be the main reason for the reduced dwell capability of the oblique target. It is worth mentioning that the above results were obtained for an unconfined silicon carbide (SiC) ceramic at the laboratory scale, which means the sizes of the rods (diameters of 0.5–2 mm) and targets were somewhat small to allow the dwell and penetration process to be examined using an X-ray flash technique. Partially because of the difficulty of obtaining X-ray images due to the larger lateral dimensions of the targets [30,32], there have been very few studies on practical engineering applications of oblique ceramic armor designed to resist the impact of large-diameter rods (diameters ≥5 mm). More importantly, the inner mechanism, the impact pressure on the target surface, and the damage to and failure of the oblique ceramic target may be quite different when large-scale rods and target obliquity are involved. The ballistic performance of the ceramic and projectile pressure attenuate with an increase in the penetrator diameter based on the cone crack growth theory [33], but improve with an increase in the ceramic’s angle of obliquity, with a long dwell time for small-scale rods impacting at a velocity of less than 1.6 km/s [31,34]. All of these oblique tests were evaluated using the depth-of-penetration (DOP) method with a semi-infinite armor steel backing. Therefore, for the practical engineering application of lightweight ceramic armor, it is necessary to study the application of oblique ceramic targets using finite backing materials designed to resist the impact of large-diameter penetrators.

The aim of this study was to investigate the ballistic performance of the designed structure by considering different impact locations of oblique targets. The oblique targets were designed to make good use of the obliquity benefit of ceramic and metallic materials. Based on the experimental results, detailed numerical simulations were conducted and analyzed to gain a better understanding of the penetration process. Furthermore, the mechanism under an oblique impact was investigated compared with that of a normal impact, where a simple layer structure was modeled using the identical line-of-sight thickness of each material used in the oblique target.

## 2. Experimental Work

### 2.1. Experimental Set-Up

**Figure 1:**
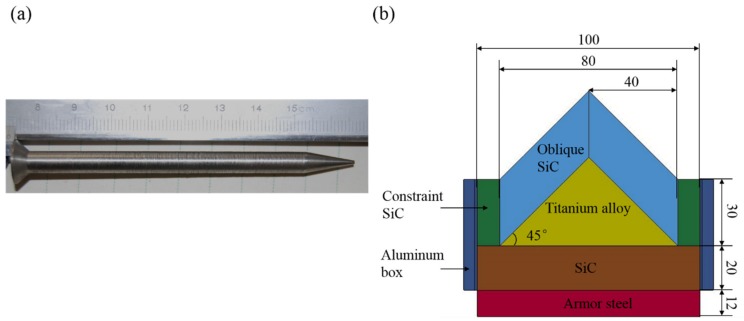
Projectile (**a**) and target configuration with unit in mm (**b**).

The tungsten-heavy-alloy (WHA) penetrator used in this study had a length of 93 mm and diameter of 6 mm, with a conical tip (a conical length of 14 mm, cone tip diameter of 1 mm, and cone angle of 10°) and conical tail (a conical length of 3 mm, cone tail diameter of 12 mm, and cone angle of 45°), as shown in Figure 1a,b which shows the cross section of the designed target configuration, which had a length of 140 mm in the third dimension. The oblique, specially shaped SiC, with a thickness of 21 mm (a line-of-sight thickness of 30 mm), was glued to triangular titanium alloy prisms with a height of 40 mm and obliquity of 45°. The backing parts consisted of layered silicon carbide (20 mm thick) and armor steel (12 mm thick) to absorb the residual energy of penetrators. The basic mechanical properties of the rod and targets are presented in Table 1. The density of the rod and target materials was measured based on Archimedes drainage method. Elastic modulus, Poisson’s ratio and Shear modulus of all materials were measured by the resonance method with the plate specimens. Tensile properties with yield strength are tested by INSTRON testing machine (Instron 5985, ITW Inc., Glenview, IL, USA). In addition, a bending test with orthogonal strain gauges for brittle silicon carbide was performed to verify the mechanical parameters.

**Figure 2:**
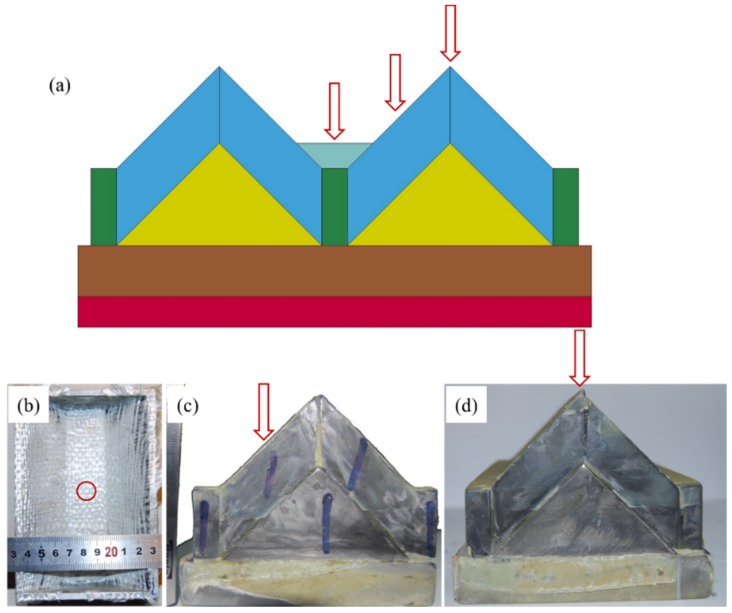
Two units of designed structure (**a**) and prepared structures except for armor steel and aluminum boxes for different impact locations: (**b**) bottom impact with ruler in cm (with aluminum boxes), (**c**) middle impact, and (**d**) top impact.

The designed target configuration could be regarded as a basic unit and extended using an array arrangement, as shown in Figure 2a. The SiC material in the region connecting the two units is 10 mm thicker to compensate for the missing mass of titanium alloy. Thus, a soft constraint using a hard aluminum box (5 mm thick) was selected for impact on just a one-unit target. All of the materials except the armor steel and aluminum boxes were integrated using epoxy resin and hot-press technology. Because of the differences in the titanium alloy thicknesses, the structure’s response to penetration was examined experimentally by considering different impact locations of the oblique targets. The prepared structures are shown in Figure 2, where Figure 2b shows a bottom impact, Figure 2c shows a middle impact, and Figure 2d shows a top impact.

The long rod projectile was accelerated by a powder gun and impacted the oblique parts at a 45° obliquity, which was normal for the backing parts. The impact velocity was nominally 1400 m/s and was captured with a laser measurement system. A witness block was used with an air gap of 50 mm just in case the rods penetrated the structures. The ballistic performance of the oblique target was then assessed based on penetrated area density $\rho_{A}$ in Equation (1) as follows:

(1) $$\rho_{A}=\rho_{SiC-1}\cdot l_{SiC-1}+\rho_{Ti6Al4V}\cdot l_{Ti6Al4V}+\rho_{SiC-2}\cdot l_{SiC-2}+\rho_{Steel}\cdot P_{r}$$

where ρ denotes the density of the materials involved, and l is the length of the materials in the impact direction. *SiC*-1 and *SiC*-2 correspond to the front oblique ceramic and backing ceramic, respectively. P_r_ is the residual depth of the penetration into the armor steel. The penetrated weight in the witness steel should also be added if it exists.

### 2.2. Experimental Results

**Figure 3:**
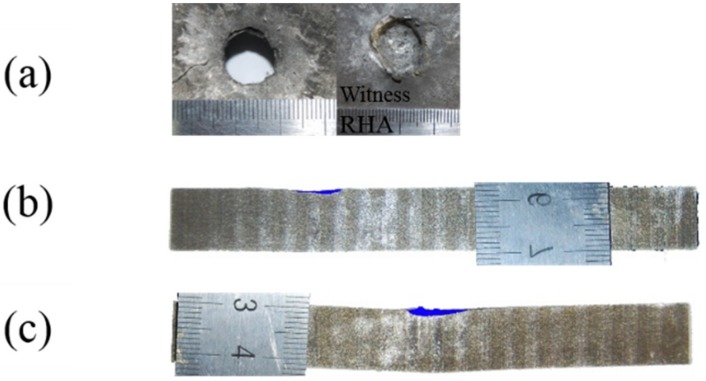
Recovered 12 mm thick armor steel with ruler in cm for different impact locations with craters in blue color: (**a**) bottom impact with witness penetration, (**b**) middle impact, and (**c**) top impact.

The experimental results for the different impact locations after penetration are summarized in Table 2. The impact velocity and residual DOP were measured. The penetrated area density data were calculated using Equation (1). The recovered images of the finite thick armor steel are presented in Figure 3. These show clear impact locations on the armor steel.

From Figure 3 and Table 2, it is observed that the ballistic responses of the ceramic armor were different at the different impact locations. Small values for the penetration and deformation of the armor steel were found for the top and middle impacts, but worse results were found for the bottom test. For the bottom impact, the silicon carbide was approximately 60 mm thick, with just 12 mm thick armor steel backing. This small thickness of backing steel did not provide enough support for the ceramics. Consequently, the long rod projectile penetrated the backing steel and was captured in the witness block, as shown in Figure 3a. This large penetration into the witness steel was included in the calculated value for the penetrated area density listed in Table 2. In the top impact, thicker titanium alloy materials were involved but did not provide better results than the middle impact. This can be partly explained through an examination of the prepared structures in Figure 2d. Because of flatness problems during the preparation, a gap (approximately 1 mm) was introduced between two ceramic columns, which weakened the ceramic’s resistance during the impact. On the other hand, the target obliquity of 0° for the top impact seemed to make no greater contribution to the ballistic resistance than the obliquity of 45° for the middle impact.

**Figure 4:**
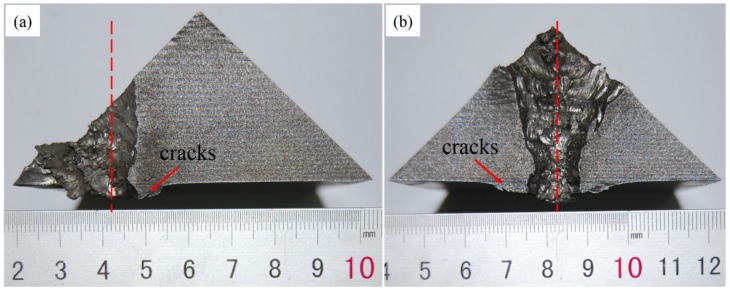
Cross sections of titanium alloys after impact: (**a**) middle impact and (**b**) top impact.

In addition, cross sections of the titanium alloy under the top and middle impacts are given in Figure 4. Both titanium materials in the interaction zone underwent serious deformation. Accordingly, a rough channel surface and some stepped cracks were observed. It should be noted that a straight channel was found under the middle impact, as shown in Figure 4a. Almost no deflection of the long rod occurred when it penetrated the front oblique ceramic and titanium parts. This means that the energy of long rods could be dispersed through tip mass erosion as the penetration proceeded. These results are in good agreement with the findings of Behner [30] for a ceramic target with a 60° obliquity. The findings are also consistent with Rosenberg’s work [3], because the 45° or 60° obliquity of the targets did not meet the obliquity requirements (≥70°) for rod deflection, although only long rods and semi-infinite armor steel were discussed in his research.

## 3. Numerical Simulations

To better understand the mechanism behind the experimental results, numerical simulations of the experiments were implemented using LS-Dyna software to reproduce the penetration and failure processes. More importantly, the experimental findings did not completely reveal the effect of target obliquity in the asymmetric structure because there were no comparisons with any other asymmetric or symmetric structures. Therefore, in addition to the simulations of the experiments, a simple layer structure with the identical line-of-sight thickness of each material used in the middle oblique impact was simulated and analyzed, with the results presented in Section 3.4.

### 3.1. Numerical Model

**Figure 5:**
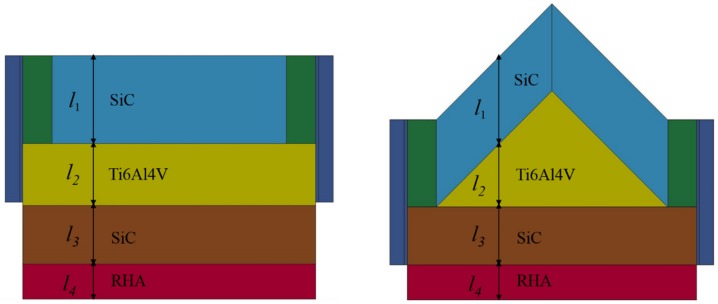
Numerical model for layer structure with 0° obliquity (**left**) and model for oblique structure with 45° obliquity (**right**). The same material thickness was used in each model, with *l*_1_ = 30 mm, *l*_2_ = 21 mm, *l*_3_ = 20 mm, and *l*_4_ = 12 mm.

Figure 5 shows the three-dimensional (3D) half models of the simple layer structure and oblique structure built for the simulations. These two models had the identical line-of-sight thickness, as measured in the middle experiment test. In other words, the front part of the layer structure contained 30 mm of silicon carbide and 21 mm of titanium alloy. The backing parts were the same as those of the oblique structure. The rod and target models were discretized using Lagrangian elements with an element size of 0.5 mm. Due to the capabilities of plasticity, stress stiffening and large strain, fully integrated 3D eight-node hexahedron solid elements were used for the rod and targets.

The Johnson-Cook (JC) model was used for the tungsten alloy, titanium alloy [35], and armor steel [36]. As listed in Table 3, the strength parameters of tungsten alloy were obtained experimentally and the erosion parameters were from the literature [37]. The Johnson-Holmquist-1 (JH-1) model was adopted for the SiC in all the cases with basic mechanical properties showed in Table 1. The test value of ceramic flexural strength was 370 ± 20 MPa, applied to the JH-1 model. The other relevant JH-1 parameters were mainly obtained from the literature [38,39,40]. The boundary conditions of the numerical simulation were the same as the practical one. In addition, there was a distal fixation at the bottom of armor steel and a symmetry constraint was defined for the half of model. The impact velocity of the rods was set to 1400 m/s according to the experimental value. A single surface contact was selected, and the simulation ignored the influence of the adhesive bond, although it was used in the experiments. The validation of the numerical model and further analyses of the rod and target performances are presented in the following section.

### 3.2. Validation of Numerical Model

Along with the experimental measurements, the simulation results are shown in Table 4. In addition to impacts at different typical locations, an RHA test (no ceramic) at an impact velocity of 1400 m/s was also carried out. The maximum penetrated weight error was 2.7% from the ballistic results obtained from experiments and simulation. The simulation data were in good agreement with the calculated penetrated area density (Equation (1)) values for the experiments, indicating that the material parameters were appropriate for the rod and target modules.

**Figure 6:**
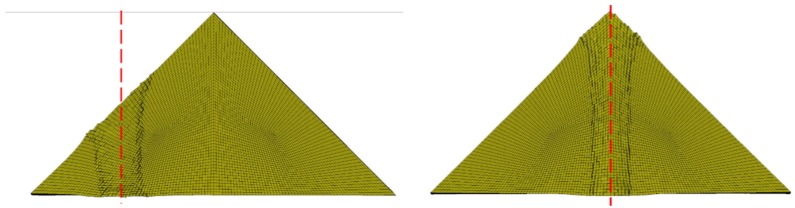
Deformation of titanium alloy in simulations of middle impact (**left**) and top impact (**right**).

A comparative analysis of the deformation of the titanium alloy are showed in Figure 6 for the middle and top impacts. The ballistic response characteristics are somewhat similar to the experimental results in Figure 4. The selected erosion model for the rod and targets were not able to reflect the eroded materials response after the erosion condition was reached. However, in experiments, large deformation of the rod and target materials led to a larger and rough impact channel.

### 3.3. Effect of Gap between Ceramics

**Figure 7:**
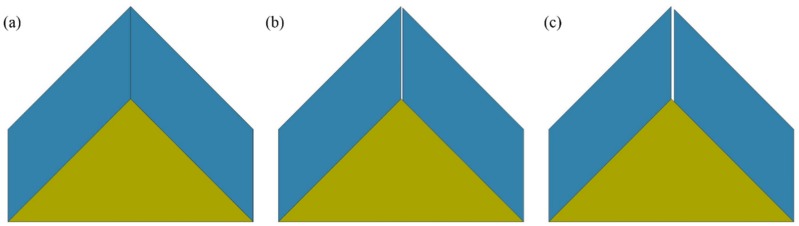
Gap size before top impact between two oblique silicon carbide pieces: (**a**) 0 mm, (**b**) 0.5 mm, and (**c**) 1 mm.

**Figure 8:**
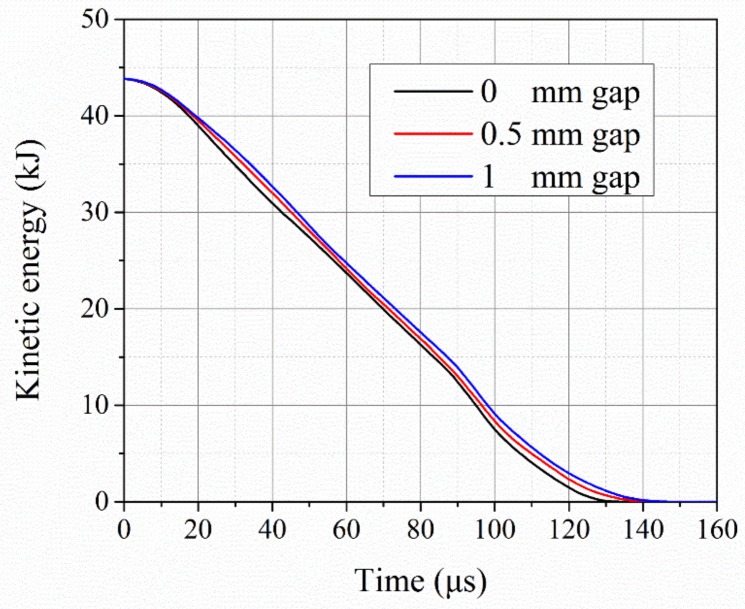
Temporal variation in kinetic energy of long rods for top impact with different gap sizes.

The experimental results for the top impact were worse than those for the middle impact even though thicker titanium alloy materials were involved. A gap that resulted from low dimensional precision between the two ceramic pieces accounted for these results. Therefore, numerical models with different gap sizes were used for further investigations. Models with the gap sizes of 0 mm, 0.5 mm, and 1 mm between the two ceramic pieces were built, as shown in Figure 7. The related kinetic energy curves for the long rods over time are given in Figure 8. It is clear that the dissipation rate of the kinetic energy decreased with an increase in the gap. Subsequently, the time for the complete erosion of rods with the 0 mm gap was approximately 131 µs, in comparison with 145 µs for the 1 mm gap. The greater interaction time resulted in a greater target penetration. Thus, it is suggested that gaps between armor structures be avoided.

### 3.4. Obliquity Effect

The ballistic responses of a simple layer structure and an oblique one for the middle test were studied for obliquity values of 0° and 45°, respectively. The simulation results for the total penetration weight are listed in Table 5. Less area density is needed in the oblique structure to defeat the long rods.

**Figure 9:**
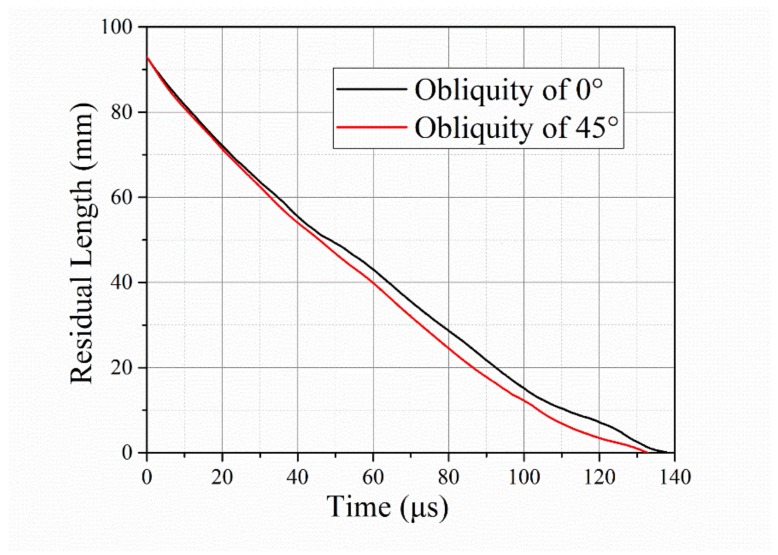
Temporal variation in rod’s residual length for target obliquity values of 0° and 45°. The beginning of time corresponds to the start of the long rod’s interaction with the target.

**Figure 10:**
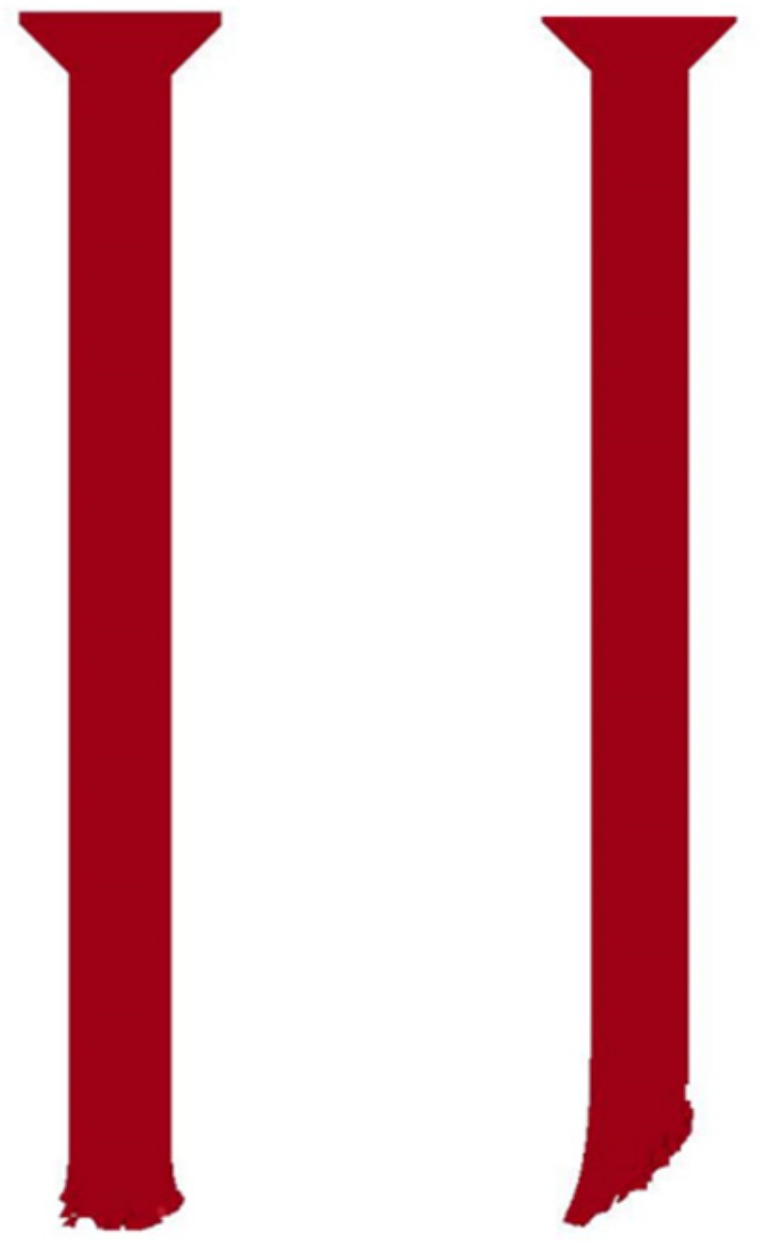
Rod image comparison at time t = 20 µs for target obliquity values of 0° (**left**) and 45° (**right**).

Figure 9 shows how the rod’s residual length changed over time. The beginning of time corresponds to the beginning of the target impact. Shorter rod lengths are observed during the whole impact process for the 45° obliquity. A rod image comparison at time t = 20 µs is presented in Figure 10. Because of the induced asymmetric force in the oblique target, the rod deformed along the target surface, forming an oblique tip.

**Figure 11:**
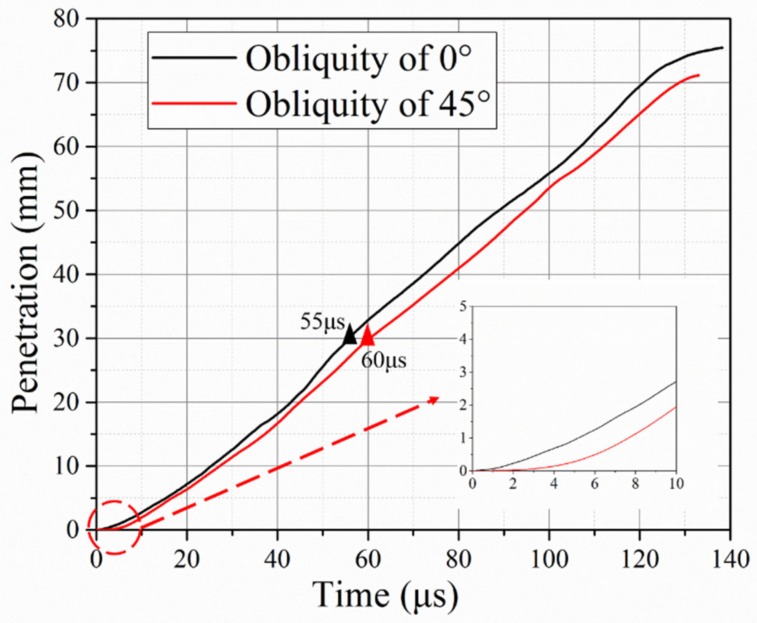
Temporal variation of rod penetration for target obliquity values of 0° and 45°.

The penetration curves over time (Figure 11) indicate an extra short dwell period for the target with 45° obliquity. The dwell time was sustained at approximately 5 µs. Subsequently, it took approximately 60 µs to penetrate through the oblique silicon carbide with a thickness of 30 mm, or nearly 55 µs for the normal impact. Based on the rod remnants (Figure 10) and penetration curves (Figure 11) over time, it can be concluded that more time and more erosion of the rods are required for penetration through the oblique ceramic than for the normal impact. Consequently, a good ballistic performance could be obtained using an oblique target.

Finally, Behner [32] concluded that the transition velocity from interface defeat to penetration was approximately 900 m/s for unconfined silicon carbide against similar rods during a normal impact. The velocity of 1400 m/s in this study must have been above the threshold, which allowed the rod to penetrate the oblique ceramic and titanium materials. The velocity of 1400 m/s in the normal impact was far more than the transition velocity (over 500 m/s) resulting in no observation of a partial dwell (Figure 11), proposed by Espinosa [41] where a short partial dwell still worked above the transition velocity range. In contrast, a short partial dwell under normal impact was observed using a fuzzy X-ray photo with a lower velocity of 1148 m/s (about 200 m/s than the transition velocity) from Lundberg’s research [33]. However, the simulation curve (Figure 11) reveals a short dwell time before the rods penetrating the ceramic in the oblique impact under the same velocity of 1400 m/s. So, it was inferred that the transition velocity was improved for oblique silicon carbide due to the positive effect of target obliquity.

**Figure 12:**
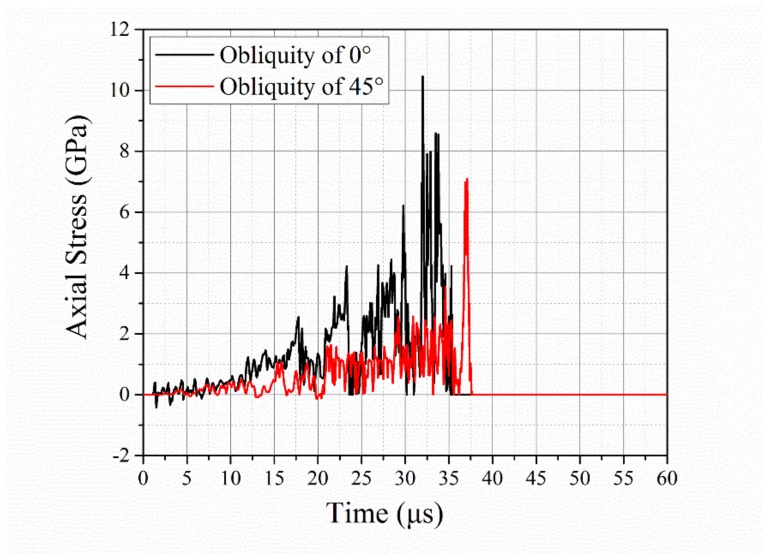
Temporal variation of axial stress in center of ceramic for target obliquity values of 0° and 45°.

**Figure 13:**
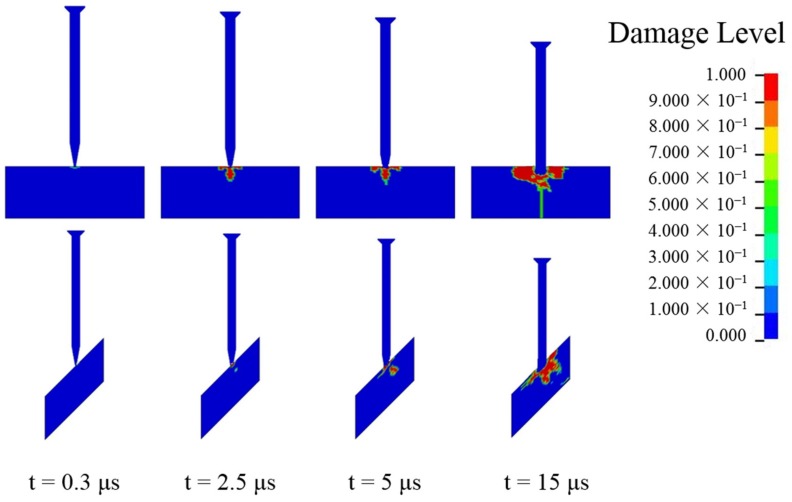
Comparison of damages to ceramic with target obliquity values of 0° (top) and 45° (bottom). At t = 5 µs, the inner damage connected to the surface damage, forming a comminution zone, resulting in the failure of the ceramic. In this regard, transition from dwell to penetration occurred.

Furthermore, a numerical analysis of the axial stress at the center of the ceramic was conducted, with the results shown in Figure 12. The axial stress was a mean value from several elements of the inner ceramic materials. The stress significantly decreased as the obliquity increased from 0 to 45°. This is usually regarded as the main reason for the obliquity benefit in ballistic resistance. Figure 13 reveals a clear difference in the failure paths of the ceramic materials. The damage starts from the surface and evolves in the radial and transverse directions under the normal impact. In contrast, inner damage shows up and develops along the ceramic thickness direction in the oblique impact. At t = 5 µs, the inner damage connected the surface damage, forming a comminution zone, resulting in the failure of the ceramic. In this regard, transition from dwell to penetration occurred, as showed in Figure 11. The damage area for the oblique impact is also much smaller at each point in time. Therefore, because of the lower axial stress and lower amount of damage to the ceramic during the initial impact, an extra dwell period occurred for the middle impact with the 45° obliquity, resulting in the improved ballistic resistance of the oblique ceramic armor.

## 4. Conclusions

An oblique ceramic armor structure was designed to defeat long rod penetrators. The structural performance at different impact locations was studied through experiments and numerical simulations. The related penetration and failure processes were analyzed, and a comparative analysis of the normal impact case was also conducted.

The influence of obliquity on the ceramic armor depended on the inner stress field and damage area of the ceramic. Once ceramic inner damage connects the surface damage, forming a comminution zone, the dwell period ends and penetration into ceramic begins. The target with 45° obliquity could decrease the penetrated areal density by 12% compared to the normal target with an identical line-of-sight materials thickness. The enhanced ballistic resistance of the oblique impact was due to an extra short time dwell period at an impact velocity of 1400 m/s.

Excellent ballistic performances were obtained for impacts at the top and middle areas of the designed structure. A lateral gap between ceramic pieces weakened the performance of the ceramic. The bottom areas of the designed structure could not defeat the long rod because of insufficient back support. There is a lot of future work to improve the performance of these weak areas by structure design, such as optimizing material size and obliquity angle or seeking more efficient materials. Meanwhile, more effective experiments and detecting technology in impact process are required for ceramic obliquity applications.

## Figures and Tables

**Table 1:** Projectile and target properties. WHA, tungsten-heavy-alloy.

Material	Density	Young’s Modulus	Shear Modulus	Poisson’s Ratio	Yield Stress
	ρ(kg/m^3^)	E/GPa	G/GPa	ν	σ/MPa
WHA		384	160	0.2	956
SiC	3160	441	183.8	0.2	-
Armor steel	7830	203	77	0.32	1100
Ti6Al4V	4430	116	44	0.317	1100
Aluminum	2700	71	27	0.32	88

**Table 2:** Experimental results.

Impact Structure	Velocity m/s	Residual DOP mm	Penetrated Area Density kg/m^2^
RHA test	1401	70.2	549.5
Bottom test	1402	20.5 *	349.1 *
Middle test	1404	1.7	263.5
Top test	1398	3.1	358.8

* including penetration of the backing steel and witness steel. No ceramic was used in the “Rolled Homogeneous Armor (RHA) test”; and the “top test,” “middle test”, and “bottom test” labels indicate the different impact locations for the oblique targets. DOP, depth-of-penetration.

**Table 3:** Johnson–Cook (JC) model constants for titanium alloy (Ti6Al4V) [35], armor steel (RHA) [36], and tungsten heavy alloy (WHA) [37].

Parameters	WHA	Ti6Al4V	RHA
Density, ρ (g/cm^3^)	17.6	4.43	7.83
Shear modulus, G (GPa)	160	44	77
Yield strength, A (GPa)	0.95	1.098	0.792
Hardening constant, B (GPa)	1.16	1.092	0.51
Hardening exponent, n	0.626	0.93	0.26
Strain rate constant, C	0.056	0.014	0.014
Reference strain rate, ${\dot{\varepsilon }}_{0}$ (s^−1^)	1	1	1
Thermal exponent, m	1	1.1	1.03
Melting temperature, $t_{m}\left(K\right)$	1723	1875	1800
Damage constant, D_1_	0	−0.09	0.05
Damage constant, D_2_	0.33	0.25	3.44
Damage constant, D_3_	−1.5	−0.5	−2.12
Damage constant, D_4_	0.042	0.014	0.002
Damage constant, D_5_	0	3.87	0.61

**Table 4:** Comparison of ballistic results obtained from experiments and simulation.

Impact Locations	Velocity m/s	Experiments		Simulation		Error	
		Residual DOP mm	Penetrated Area Density kg/m^2^	Residual DOP mm	Penetrated Area Density kg/m^2^	Residual DOP mm	Penetrated Area Density kg/m^2^
RHA test	1401	70.2	549.5	68.3	534.9	2.7%	2.7%
Bottom	1402	20.5 *	349.1 *	19.6 *	342.4 *	1.9%	4.2%
Middle	1404	1.7	263.5	1.9	265.4	−0.7%	−14.3%
Top	1398	3.1	358.8	2.0	350.4	2.3%	34.7%

* including penetration of the backing steel and witness steel. No ceramic was used in the “RHA” test; and the “top test,” “middle test,” and “bottom test” labels indicate the different impact locations for the oblique targets.

**Table 5:** Simulation results of normal and oblique impact.

Impact Structure	Velocity m/s	Penetrated Area Density kg/m^2^
Normal impact	1400	285.9
Oblique impact	1400	252.1
